# Echocardiography Report Translation and Inference Based on Parameter-Efficient Fine-Tuning of LLaMA Models

**DOI:** 10.3390/diagnostics16081223

**Published:** 2026-04-20

**Authors:** Hsin-Ta Chiao, Wei-Wen Lin, Shang-Yang Tseng, Yu-Cheng Hsieh, Chao-Tung Yang

**Affiliations:** 1 Department of Computer Science, Tunghai University, Taichung 407224, Taiwan; josephchiao@thu.edu.tw (H.-T.C.); g12350038@go.thu.edu.tw (S.-Y.T.); 2 Cardiovascular Center, Taichung Veterans General Hospital, Taichung 407219, Taiwan; ychsieh@vghtc.gov.tw; 3 Department of Post-Baccalaureate Medicine, College of Medicine, National Chung Hsing University, Taichung 402202, Taiwan; 4 Department of Life Science, Tunghai University, Taichung 407224, Taiwan; 5 Department of Medical Research, Taichung Veterans General Hospital, Taichung 407219, Taiwan; 6 Department of Medical Research, Institute of Clinical Medicine, National Yang Ming Chiao Tung University, Taipei 112304, Taiwan; 7 Research Center for Smart Sustainable Circular Economy, Tunghai University, No. 1727, Sec. 4, Taiwan Boulevard, Taichung 407224, Taiwan; 8 Department of Medical Research, Kuang Tien General Hospital, No. 127, Sec. 7, Xiangshang Rd. Shalu Dist., Taichung 433004, Taiwan

**Keywords:** large language models, QLoRA, echocardiography reports, natural language generation, fine-tuning

## Abstract

**Background/Objectives:** Echocardiography reports are essential diagnostic tools, but their complexity and specialized English terminology frequently hinder comprehension for non-specialists and patients. This study addresses these accessibility gaps by developing a resource-efficient large language model (LLM) system designed to translate and summarize English echocardiography results into Traditional Chinese. **Methods:** To overcome significant hardware constraints, we utilized Quantized Low-Rank Adapter (QLoRA) techniques and the Unsloth acceleration framework to fine-tune LLaMA-3.2-1B and LLaMA-3.2-3B-Instruct models on a single mid-tier GPU. The system employs a dual-stage inference architecture: the first stage provides technical medical translation for clinicians, while the second stage generates simplified, patient-centric educational summaries to enhance health literacy. **Results:** Evaluation across multiple metrics, including BLEU, ROUGE, METEOR, and Perplexity, demonstrated that the LLaMA-3.2-3B-Instruct model with the AdamW 8-bit optimizer achieved the most stable validation performance, excelling in semantic coherence and structural consistency. A preliminary qualitative error analysis conducted in the Discussion section further identified clinical nuances, such as terminology simplification and minor hallucinations, underscoring the critical necessity of a Human-in-the-Loop verification procedure. **Conclusions:** These findings validate the feasibility of deploying cutting-edge medical AI in resource-limited clinical environments. While the results reflect validation-only performance on a specialized dataset, the platform offers a scalable foundation for enhancing clinical decision support and health literacy through accessible, automated medical text processing.

## 1. Introduction

Large language models (LLMs) have demonstrated exceptional capabilities in language understanding and generation in recent years, significantly advancing the development of intelligent applications across various domains in the field of natural language processing (NLP). However, as model sizes continue to grow, the extensive memory and computational resources required for traditional full-parameter fine-tuning have become a major challenge for model deployment and practical use [1]. This issue is particularly prominent in the medical domain, where tasks such as translating and generating inferences from structured and domain-specific texts—such as echocardiography reports—demand not only high precision in language processing but also efficiency in resource usage and inference quality.

To address the limitations of fine-tuning large models under resource-constrained environments, the Quantized Low-Rank Adapter (QLoRA) technique has emerged. By combining 4-bit quantization with low-rank adapter training, QLoRA significantly reduces memory consumption while retaining model performance, enabling efficient fine-tuning [1,2]. Furthermore, the Unsloth framework systematically optimizes the QLoRA training pipeline through enhanced memory management and gradient accumulation mechanisms, allowing large language models to be fine-tuned effectively on a single GPU. This approach has demonstrated promising results in multiple domains, including healthcare, chemistry, and sentiment analysis [3,4,5].

Echocardiography is a critical diagnostic tool in cardiology, widely used for the evaluation of cardiovascular diseases. However, its associated reports are often written in English with complex structure and dense medical terminology, making them difficult to interpret for non-cardiologist physicians and general healthcare personnel. Accurately translating these echocardiography reports into Traditional Chinese and generating simplified summaries with treatment suggestions not only enhances the accessibility and readability of medical information but also improves communication efficiency in clinical settings, offering significant practical value.

Traditionally, training large language models (LLMs) for domain-specific tasks requires substantial memory and computational resources, posing a major barrier for many healthcare institutions and research teams due to the high costs of deployment and infrastructure. With the emergence of parameter-efficient fine-tuning techniques, Quantized Low-Rank Adapter (QLoRA) has provided a promising solution by integrating 4-bit quantization with low-rank updates, significantly reducing training costs while maintaining model performance [1,2]. In addition, the Unsloth framework further optimizes the QLoRA training process, enabling effective fine-tuning of large models on a single GPU and expanding the feasibility of LLM applications under resource-constrained conditions [3,4,5].

This study identifies and addresses several critical gaps at the intersection of cardiology, healthcare accessibility, and resource-efficient artificial intelligence:

- **Linguistic and Specialized Knowledge Barrier:** Echocardiography reports are traditionally written in English with dense, technical terminology, creating a significant information gap for non-specialist physicians and the general public in non-English speaking regions. This study fills this gap by providing a dual-stage system that translates reports into formal Traditional Chinese and generates accessible, patient-centric summaries.
- **Deployment in Resource-Constrained Environments:** While state-of-the-art medical LLMs exist, they often require extensive computational infrastructure (e.g., TPU clusters) that is inaccessible to small clinics [6]. This research fills a technical deployment gap by validating that high-quality clinical text generation is feasible on a single mid-tier GPU using QLoRA and the Unsloth framework [1].
- **Specialization for Structured Diagnostic Reports:** Many existing medical models are optimized for general dialogue rather than the structured document processing required for diagnostic records [7]. This paper addresses this domain-specific processing gap by ensuring the semantic integrity of cardiac parameters, such as valvular dysfunction and ventricular function, during the translation and summarization process.
- **Integration of Clinical Workflow Tasks:** Existing research often treats translation and summarization as isolated tasks. This study fills a workflow gap by proposing a two-stage inference architecture that creates a seamless information pipeline from raw English reports to actionable clinical insights for both professionals and laypeople.

This study aims to integrate Quantized Low-Rank Adapter (QLoRA) technology with the Unsloth acceleration framework to develop a dual-stage inference system based on large language models (LLMs) for the translation and summarization of echocardiography reports, specifically under limited hardware resource conditions. By efficiently fine-tuning LLMs, the system is designed to accurately translate English echocardiography reports into formal Traditional Chinese medical texts, and subsequently generate concise and comprehensible diagnostic summaries and treatment suggestions. This is intended to enhance the accessibility and usability of such reports for non-cardiologist physicians and the general public. To achieve this goal, the objectives of this paper are listed as follows:

1. QLoRA is employed to perform parameter-efficient fine-tuning in low-memory environments, ensuring both training and inference performance remain stable and reliable. In parallel, the Unsloth framework is applied to further optimize the QLoRA fine-tuning pipeline by improving memory management and gradient accumulation, thereby accelerating training and lowering hardware requirements. The system adopts a two-stage inference architecture: the first stage focuses on translating the English echocardiography report, while the second stage generates simplified summaries and inference-based suggestions based on the translated text, balancing clinical applicability and the dissemination of medical information.
2. To ensure practicality, the system is designed to operate on a single mid-tier GPU (e.g., NVIDIA Tesla T4), making it more feasible for deployment in non-academic and small clinical settings. This configuration enables hospitals and clinics without access to high-end computing infrastructure to benefit from large language model capabilities. Furthermore, this study emphasizes the preservation of medical accuracy and semantic integrity in both the translation and summarization processes, particularly in handling structured and domain-specific terminology found in cardiology reports.
3. The selection of LLaMA models for fine-tuning is motivated by their open-access architecture, strong generalization ability, and availability of high-quality instruction-tuned checkpoints. By combining these models with quantization and low-rank adaptation techniques, the system significantly reduces the number of trainable parameters and VRAM usage while preserving generation quality. This setup enables faster experimentation and lowers the technical barrier for future researchers or developers working on domain-specific applications in low-resource settings.
4. To evaluate the system’s effectiveness, the study adopts multiple automatic evaluation metrics—BLEU, ROUGE, METEOR, and Perplexity—each capturing different aspects of translation fluency, semantic coverage, and model fluency. This multi-metric design ensures a comprehensive assessment across both surface-level and deeper semantic qualities. In the future, the system can be extended to support other medical domains such as radiology, pathology, or general patient discharge summaries by retraining on domain-specific datasets.

## 2. Methods

### 2.1. Large Language Models, LLMs

Large language models (LLMs) have rapidly emerged in recent years as a core technology driving innovation in natural language processing (NLP) and a wide range of cross-domain applications in the field of artificial intelligence. Through pretraining on large-scale corpora, LLMs are capable of deep language understanding, reasoning, and generation abilities [8]. Yuan et al. [9] pointed out that as AI technologies continue to advance, the potential of LLMs in the medical domain is becoming increasingly evident, not only supporting medical education but also aiding clinical decision-making, research facilitation, and workflow automation.

Zheng et al. [8] conducted a systematic review of the current applications of LLMs in medicine, highlighting their utility in tasks such as electronic health record (EHR) summarization, medical literature retrieval, clinical decision support, and patient education. These applications demonstrate the potential of LLMs to improve healthcare efficiency and the quality of patient care. Yuan et al. [9] further explored the integration of multimodal data—such as medical imaging, genomic information, and EHRs—into LLM applications, emphasizing the importance of developing multimodal large models to accommodate the diversity and complexity of medical data.

In the context of medical education, Lucas et al. [10] proposed that LLMs have the potential to support medical student learning by simulating clinical scenarios and providing real-time feedback, thereby enhancing diagnostic reasoning and clinical decision-making skills. Their systematic review indicated that models like ChatGPT (GPT-5 series) perform well on standardized exams, but careful design of educational use cases is necessary to avoid overreliance on AI tools and to preserve students’ critical thinking development.

From the perspective of medical AI system development, Mao et al. [11] emphasized the critical role of LLMs in advancing intelligent healthcare systems. They introduced the concept of “multi-dimensional challenges for medical LLMs,” suggesting that future systems must address multimodal integration, inter-model collaboration, cultural adaptability, and ethical responsibility to achieve sustainability and reliability in clinical settings.

Despite their promise, current applications of LLMs in healthcare still face numerous challenges. Chen et al. [12] pointed out that although LLMs perform at near-human levels on standardized exams, they continue to struggle with clinical reasoning, factual accuracy, and human-like communication. They emphasized the need for incorporating multimodal data and adopting human-centered design principles to enhance the practical effectiveness of LLMs in real-world healthcare environments.

With their advanced capabilities in language understanding, reasoning, and generation, LLMs are profoundly reshaping medical information processing, clinical decision support, and medical education. However, responsible and effective deployment of LLMs in the medical domain will require continued efforts to address challenges such as accuracy, bias mitigation, ethical compliance, and rigorous clinical validation.

Building on this foundation, recent studies have begun to explore how large language models can be further enhanced through multimodal integration. For example, Niu et al. [13] presented a comprehensive survey on Multimodal LLMs (MLLMs), identifying their potential to process both text and images in clinical domains such as radiology, dermatology, and electronic health records. The authors highlighted representative systems including LLaVA-Med, SkinGPT-4, and Med-Flamingo, while also outlining major challenges such as modality alignment, data availability, and interpretability.

Complementing this, Agbareia et al. [14] empirically evaluated GPT-4o and Claude Sonnet 3.5 on diagnostic tasks using real patient cases with both textual and image inputs. Their findings showed that although LLMs can benefit from image information, their ability to interpret and integrate visual data remains limited compared to human physicians.

These studies reinforce the growing importance of multimodal reasoning in medical AI while also underscoring the current technical limitations. In contrast, this study focuses specifically on language-centered tasks—namely the translation and summarization of echocardiography reports—where high performance can be achieved even under constrained resources, without relying on large-scale multimodal data.

### 2.2. LLaMA

Large language models (LLMs) have achieved remarkable breakthroughs in the field of natural language processing. Among them, the LLaMA (large language model Meta AI) series proposed by Meta AI has played a critical role as an open-source alternative, significantly lowering development costs and promoting research openness [15]. With its multilingual training architecture and scalable parameter sizes ranging from 7B to 65B, the LLaMA series provides the developer community with a highly adaptable and competitive foundation model. It has been widely adopted in downstream tasks such as instruction tuning, domain adaptation, and knowledge augmentation.

While the original LLaMA models were trained primarily on general-domain data, their performance remains limited when applied to highly specialized domains such as medicine. To address this gap, researchers have proposed several LLaMA-based adaptations for medical applications. Li et al. [7] introduced ChatDoctor, which fine-tunes the LLaMA-7B model using 100,000 real patient-doctor dialogues with instruction-following and domain knowledge enhancement techniques. In addition, they integrated an automated knowledge retrieval mechanism that allows the model to reference online medical databases in real time, substantially improving its accuracy in answering queries related to emerging diseases and new medications.

Xie et al. [16] developed the Me-LLaMA series, based on continued pretraining and instruction tuning of the LLaMA2 model. They constructed a large-scale medical training dataset containing 129 billion characters and 210,000 instruction samples, and proposed a multi-task evaluation benchmark called MIBE (Medical Instruction Benchmark Evaluation). Experimental results showed that Me-LLaMA-Chat outperformed other open-source medical LLMs of similar scale and even surpassed GPT-4 in more than 50% of the evaluated tasks.

In Chinese medical applications, Wang et al. [15] proposed the HuaTuo model, demonstrating LLaMA’s adaptability to multilingual healthcare scenarios. They incorporated both structured and unstructured knowledge from CMeKG (Chinese Medical Knowledge Graph) and generated over 8000 synthetic medical instruction samples for fine-tuning. The team also introduced a comprehensive evaluation metric called SUS (Safety, Usability, Smoothness) to assess model responses from multiple perspectives, including safety, professionalism, and fluency. Results indicated that HuaTuo outperformed the base LLaMA model and other Chinese dialogue systems in terms of knowledge accuracy and language naturalness.

As a leading open-source LLM, LLaMA has demonstrated strong scalability and task adaptability through a variety of domain-specific fine-tuning efforts, including ChatDoctor, Me-LLaMA, and HuaTuo. Future research may further explore optimization strategies for continued pretraining and instruction tuning to enhance the practicality and reliability of the LLaMA series in high-risk, professional domains such as healthcare.

### 2.3. Quantized Low-Rank Adapter (QLoRA)

As fine-tuning techniques for large language models (LLMs) continue to evolve, Quantized Low-Rank Adapter (QLoRA) has emerged as a highly influential approach, especially in resource-constrained environments. QLoRA was proposed to combine low-rank adaptation (LoRA) with quantization techniques, aiming to significantly reduce memory usage and computational cost during training while preserving model performance.

According to the original framework proposed by Dettmers et al. [1], QLoRA introduces three key innovations. First, it employs 4-bit NormalFloat (NF4) quantization to compress pretrained model weights to 4-bit representations, effectively lowering memory footprint with minimal performance degradation. Second, it applies double quantization to further compress quantization constants for more efficient storage. Third, it integrates paged optimizers with unified memory management to enable fine-tuning of models with up to 65 billion parameters on a single 48GB GPU.

In the medical domain, QLoRA has demonstrated strong potential. Schreiber et al. [2] applied QLoRA to tasks such as protein binding site prediction and post-translational modification (PTM) detection, finding that QLoRA improves generalization performance on small datasets. Compared to traditional full fine-tuning, QLoRA’s combination of low-rank updates and 4-bit quantization helped reduce overfitting while maintaining excellent predictive accuracy using fewer hardware resources.

Sukeda et al. [17] investigated QLoRA in Japanese medical question answering (Medical QA) through the development of the JMedLoRA model. Their experiments compared LoRA and QLoRA across different model sizes, demonstrating that QLoRA can effectively integrate domain-specific medical knowledge in large models such as LLaMA2-70B with lower computational cost. The study also noted that in a few-shot inference scenarios, QLoRA’s impact on single-shot reasoning performance requires careful evaluation.

As QLoRA adoption grows, researchers have begun exploring its limitations and proposing improvements. Rajabzadeh et al. [18] introduced QDyLoRA (Quantized Dynamic LoRA), which incorporates dynamic rank selection to support multiple rank configurations within a single fine-tuning process. This approach enhances QLoRA’s flexibility and efficiency in multi-task and multi-deployment scenarios while avoiding the redundancy of maintaining separate QLoRA checkpoints for each rank.

Lawton et al. [19] proposed QuAILoRA (Quantization-Aware Initialization for LoRA), which focuses on mitigating quantization error during the initialization of LoRA parameters. Through a calibration-based strategy, QuAILoRA minimizes quantization-induced errors without increasing memory overhead. Experimental results showed that QuAILoRA can deliver inference performance close to that of 8-bit or even 16-bit models while retaining the compactness of 4-bit quantization, highlighting its reliability and effectiveness.

As an innovative technique combining quantization and low-rank adaptation, QLoRA has had a profound impact on language model fine-tuning and has shown excellent potential in professional domains such as healthcare. As the technique continues to mature, future research will likely focus on improving QLoRA’s flexibility during inference, stability in initialization, and adaptability to low-resource environments.

### 2.4. Unsloth

As large language models (LLMs) are increasingly adopted across diverse domains, traditional fine-tuning methods have begun to reveal limitations, including excessive memory consumption, slow training speed, and high hardware requirements. To address these bottlenecks, the Unsloth framework was introduced as an optimization tool for fine-tuning large models in resource-constrained environments.

The core objective of Unsloth is to improve the efficiency and stability of the fine-tuning process. Specifically, Unsloth enhances the underlying Hugging Face Transformers models by integrating 4-bit quantization (bnb-4bit), Low-Rank Adaptation (LoRA), and efficient gradient accumulation strategies. This combination enables effective fine-tuning of large models even on mid- to low-end GPUs. In the study by Dai et al. [3] focused on traditional Chinese medicine, Unsloth was used in conjunction with LLaMA3 and Phi-3 series models, successfully achieving low-memory, single-GPU fine-tuning, demonstrating the framework’s versatility in medical natural language processing tasks.

In the domain of scientific computing, Ma et al. [4] applied the Unsloth framework to fine-tune the Generalized Vision Information Model (GVIM) for cheminformatics applications. Their work involved models such as LLaMA3-8B and Gemma-9B, and achieved successful training of models exceeding 8 billion parameters on a single GPU. The results indicated that Unsloth can significantly reduce resource consumption while maintaining high model performance. The authors further noted that Unsloth’s combination of memory release mechanisms and efficient gradient accumulation enables training tasks previously requiring multiple GPUs to be completed on a single device.

Unsloth also demonstrated excellent performance in Sentiment Intensity Regression (SIR) tasks. In the experiments conducted by Diefan Lin et al. [5] using the SemEval 2017 and 2018 datasets, Unsloth was combined with LoRA to fine-tune the LLaMA3-8B model on a Tesla P100 GPU with only 16 GB of memory. The results showed significant improvements in evaluation metrics such as MAE, MSE, and Pearson correlation coefficient under low-resource conditions. The fine-tuned models outperformed baseline systems such as RoBERTa, Gemma, and EmoLLM. The study also demonstrated that by integrating Unsloth’s memory optimization with LoRA, overfitting during large model fine-tuning can be effectively mitigated, leading to better downstream task performance.

As an efficient and resource-friendly fine-tuning framework, Unsloth has shown remarkable performance across various applications, including traditional medicine, cheminformatics, and sentiment intensity regression. With its integration of quantization techniques, LoRA adapters, and dynamic memory management, Unsloth provides a practical solution for large model fine-tuning under limited hardware conditions. Its application scope and technical depth are expected to expand further across professional domains in the near future.

### 2.5. QLoRA: Background and Core Concepts

QLoRA is a fine-tuning optimization technique designed for large language models (LLMs), integrating two core concepts: weight quantization and low-rank adaptation. Its primary objective is to significantly reduce VRAM usage during training while preserving the output quality of the fine-tuned model.

Under the QLoRA framework, the original model weights are quantized into low-bit representations (typically 4-bit), dramatically reducing the model’s storage and computational demands. After quantization, the main model weights remain frozen during training and are no longer updated. Instead, small trainable low-rank matrices are inserted into specific submodules—such as the query, key, and value matrices within the attention mechanism—to serve as the source of weight adaptation.

Compared to traditional LoRA methods, QLoRA further incorporates quantization optimizations, reducing memory consumption to roughly one-fourth to one-eighth of that required by standard fine-tuning. This substantial reduction makes it feasible to fine-tune large models even on mid-range hardware. The design is particularly well-suited for this study’s dual-model training scenario, enabling successful fine-tuning of LLMs with 1B to 3B parameters on a single GPU.

### 2.6. Mathematical Formulation of QLoRA

The mathematical foundation of QLoRA consists of two primary steps. First, the original weight matrix $W_{0}\in R^{d\times k}$ is quantized into low-bit representations. Then, a trainable low-rank residual matrix ΔW is introduced on top of the quantized weights. The quantization process applies a quantization function Q(·) to the original weights to obtain the quantized weight matrix $W_{q}$ as follows:

(1) $$W_{q}=Q\left(W_{0}\right)$$

In this step, each original floating-point parameter (typically 16-bit or 32-bit) is compressed to a 4-bit or lower representation, often using the NormalFloat 4-bit format to minimize information loss and maintain computational stability.

The quantized weight matrix $W_{q}$ remains frozen and is not updated during training. To enable task-specific adaptability on top of the quantized foundation, QLoRA introduces a trainable low-rank residual matrix ΔW into designated submodules. The effective weight after fine-tuning is thus expressed as follows:

(2) $$W^{\prime }=W_{q}+\Delta W$$

The residual matrix ΔW is decomposed into the product of two smaller matrices *A* and *B*:

(3) ΔW=A×B

This design significantly reduces the number of additional trainable parameters. For example, when d=k=4096 and the rank r=16, the number of new parameters amounts to only 0.8% of the full model’s original parameter count, achieving considerable efficiency in memory and computation.

During backpropagation, only the weights of *A* and *B* are updated, while the quantized base weights $W_{q}$ remain frozen. As the number of trainable parameters is minimal, this setup dramatically reduces memory consumption and computational overhead while preserving the model’s ability to adapt outputs for specific tasks. This architecture enables efficient fine-tuning of large-scale language models even in resource-constrained environments, maintaining strong task performance with minimal training cost.

### 2.7. Evaluation Metrics Overview

To comprehensively evaluate the generation quality of the fine-tuned large language models in the tasks of translating and summarizing English echocardiography reports, this study adopts BLEU, ROUGE, METEOR, and Perplexity as the primary evaluation metrics. This section introduces the calculation principles and practical implications of each metric.

BLEU (Bilingual Evaluation Understudy) measures the degree of overlap between generated text and reference text at the n-gram level. It first computes the precision of matched n-grams (such as 1-gram and 2-gram) between the generated and reference outputs, takes a weighted geometric average of these precisions, and applies a brevity penalty to discourage overly short outputs. BLEU emphasizes precision, making it effective for evaluating the local coherence and accuracy of translations. It is one of the most widely used metrics in machine translation and natural language generation. This study primarily reports BLEU-4, which considers up to 4-gram matches.

BLEU evaluates the overlap of n-grams between the generated output and the reference translation. The core formula is as follows:

(4) $$\mathrm{BLEU}=\mathrm{BP}\cdot \exp\left(\sum_{n=1}^{N}w_{n}\log p_{n}\right)$$

where:

- $p_{n}$ is the modified precision for n-grams of order *n*;
- $w_{n}$ is the weight assigned to each n-gram (commonly uniform).
- BP is the brevity penalty defined as follows:

(5) $$\mathrm{BP}=\left\{\begin{array}{cc} 1 & \mathrm{if}\;c>r\\ \exp\left(1-\frac{r}{c}\right) & \mathrm{if}\;c\leq r \end{array}\right.$$

with *c* being the length of the generated sentence and *r* the length of the reference sentence.

ROUGE (Recall-Oriented Understudy for Gisting Evaluation), in contrast, emphasizes recall, evaluating how much of the reference content is covered by the generated text. This study uses three ROUGE variants: ROUGE-1, ROUGE-2, and ROUGE-L, corresponding to unigram, bigram, and longest common subsequence (LCS) overlap, respectively. ROUGE-1 and ROUGE-2 assess local semantic consistency, while ROUGE-L captures global sentence structure similarity. ROUGE is particularly suitable for summarization and generative tasks, where coverage of reference content is critical.

ROUGE-N measures the recall of n-grams. The formula is as follows:

(6) $$\mathrm{ROUGE}\text{-}N=\frac{\sum_{\mathrm{ref}\in \mathrm{Ref}}\sum_{{\mathrm{gram}}_{n}\in \mathrm{ref}}\min\left({\mathrm{Count}}_{\mathrm{match}}\left({\mathrm{gram}}_{n}\right),{\mathrm{Count}}_{\mathrm{cand}}\left({\mathrm{gram}}_{n}\right)\right)}{\sum_{\mathrm{ref}\in \mathrm{Ref}}\sum_{{\mathrm{gram}}_{n}\in \mathrm{ref}}{\mathrm{Count}}_{\mathrm{ref}}\left({\mathrm{gram}}_{n}\right)}$$

where:

- ${\mathrm{gram}}_{n}$ denotes an n-gram;
- ${\mathrm{Count}}_{\mathrm{match}}$ is the number of overlapping n-grams between candidate and reference;
- This version focuses on recall; F1-based ROUGE can be extended accordingly.

METEOR (Metric for Evaluation of Translation with Explicit ORdering) combines precision and recall, and introduces mechanisms such as stemming and synonym matching to better capture semantic alignment. It also penalizes fragmentation in matching segments, thereby assessing both fluency and syntactic coherence. Compared to BLEU and ROUGE, which focus on surface-level n-gram matches, METEOR is more sensitive to semantic similarity, making it especially appropriate for small datasets or tasks requiring high semantic fidelity.

METEOR combines unigram precision, recall, and alignment fragmentation. First, compute precision and recall:

(7) $$P=\frac{m}{w_{\mathrm{hyp}}},\;R=\frac{m}{w_{\mathrm{ref}}}$$

where:

- *m* is the number of matched unigrams;
- $w_{\mathrm{hyp}}$ and $w_{\mathrm{ref}}$ are the lengths of the hypothesis and reference, respectively.

Then, compute the harmonic mean:

(8) $$F_{\mathrm{mean}}=\frac{10PR}{R+9P}$$

And apply the penalty term:

(9) $$\mathrm{Penalty}=0.5{\left(\frac{c}{m}\right)}^{3},\;\mathrm{METEOR}=F_{\mathrm{mean}}\cdot \left(1-\mathrm{Penalty}\right)$$

where *c* is the number of chunks (consecutively matched segments).

Perplexity measures how well a language model predicts a test set. It reflects the model’s ability to anticipate the next word given the previous context. A lower perplexity indicates stronger command over language structure, typically resulting in more fluent and natural outputs. As an intrinsic metric that does not rely on reference answers, perplexity provides an independent measure of a model’s language modeling capability.

Perplexity evaluates the predictive capability of a language model:

(10) $$\mathrm{Perplexity}=\exp\left(-\frac{1}{N}\sum_{i=1}^{N}\log p\left(w_{i}\right)\right)$$

where:

- *N* is the total number of words in the test corpus;
- $p(w_{i})$ is the predicted probability of the *i*-th word.

A lower perplexity indicates better language modeling performance. This study does not adopt semantic similarity evaluation metrics such as BERTScore and Sentence Transformer (ST), primarily due to the lack of domain adaptation of their underlying embedding models for medical texts. Although BERTScore and ST offer semantic-level comparison capabilities, the language models they rely on are generally trained on general-domain corpora and do not adequately capture clinical terminologies or syntactic structures. As a result, these methods often produce inaccurate or overly optimistic similarity scores when applied to medical texts, failing to provide clinically meaningful evaluation results.

The texts addressed in this study exhibit a high degree of structural complexity and domain-specific medical language. Accuracy and consistency of expression are the primary evaluation goals. Therefore, this study employs BLEU, ROUGE, and METEOR—metrics that effectively measure linguistic correctness and surface-level semantic coverage to assess the quality of generated outputs in translation and summarization tasks. In particular, METEOR incorporates certain semantic matching mechanisms, including stemming and synonym recognition, which help compensate for the limitations of BLEU and ROUGE in capturing deeper semantic relations. Collectively, these metrics provide a sufficient and reliable evaluation framework for the objectives of this study.

### 2.8. Related Works

Recent advancements in medical large language models (LLMs) have introduced several specialized systems designed for clinical question answering, biomedical knowledge extraction, and general medical NLP tasks. Among the most notable are ChatDoctor, BioGPT, and Med-PaLM, each of which demonstrates distinct model designs and application scopes.

ChatDoctor [7] is a medical dialogue model based on LLaMA-7B, fine-tuned using over 100K real-world doctor–patient interactions. It supports multi-turn clinical conversations and incorporates autonomous knowledge retrieval from external sources. While ChatDoctor excels in interactive medical consultation, its architecture is optimized for dialogue coherence rather than structured document processing or generation tasks.

BioGPT [20] is a biomedical generative model pre-trained on 15 million PubMed abstracts using the GPT-2 architecture. It shows strong performance in biomedical named entity recognition, relation extraction, and PubMedQA. However, BioGPT is trained only on scientific literature, without task-specific fine-tuning for clinical documentation tasks such as summarization or translation. Furthermore, it lacks instruction alignment, limiting its adaptability to structured generation.

Med-PaLM [6] represents a state-of-the-art medical QA system built on PaLM and PaLM 2. Through extensive instruction tuning and retrieval augmentation, Med-PaLM achieved 86.5% accuracy on USMLE-style multiple-choice exams and was rated by clinicians as producing clinically acceptable answers in over 90% of test cases. However, its large-scale infrastructure requires TPU clusters and extensive computational resources, posing deployment challenges in low-resource settings.

According to Clément Christophe et al. [21], fine-tuning typically involves additional task-specific training on top of a pretrained large-scale model to adapt it to the unique data distribution and linguistic patterns of the target task. This process effectively transforms the general-purpose knowledge encoded in the model into task-specific capabilities, allowing LLMs to perform better in highly specialized domains such as healthcare, law, and education.

Jiawei Chen et al. [22] further emphasized the importance of fine-tuning in the context of medical visual-language models (Med-VLMs). Due to the highly specialized, structurally complex, and limited nature of medical data, directly applying a general pretrained model often fails to yield satisfactory results. By employing appropriate fine-tuning strategies, models can better learn the correspondence between medical images and textual descriptions, thereby improving diagnostic accuracy and inferential performance.

In their work on medical image classification, Ana Davila et al. [23] highlighted that the choice of fine-tuning strategy has a significant impact on model effectiveness. Through a systematic comparison of different methods, they found that full-parameter fine-tuning generally achieves the best results. However, in low-resource settings or when data is limited, well-designed partial fine-tuning approaches can also deliver competitive performance, suggesting that fine-tuning strategies should be selected flexibly based on the specific task and constraints.

According to Savage et al. [24], Supervised Fine-Tuning (SFT) typically uses Maximum Likelihood Estimation (MLE) as the training objective. It fine-tunes the model parameters by minimizing the difference between the predicted outputs and ground-truth labels, thereby improving the model’s ability to map inputs to appropriate responses. This approach is particularly well-suited for tasks with clearly defined input-output formats and consistent standards, such as classification, question answering, and summarization.

In the medical domain, Khan et al. [25] emphasized the importance of fine-tuning for adapting self-supervised medical imaging models to downstream clinical tasks. Given the limited availability and highly specialized nature of medical data, relying solely on pretrained models often leads to suboptimal performance. SFT is therefore essential for refining the model’s ability to capture lesion characteristics, diagnostic classification standards, and clinical context. Their findings also suggest that the quantity and quality of labeled data used for SFT can significantly impact the final model performance across different tasks.

In their work on medical image classification, Mao et al. [26] demonstrated that SFT not only improves performance on diagnostic tasks, but that carefully designed fine-tuning strategies—such as consistent input encoding and masking techniques—can also enhance model inference stability and computational efficiency. Their proposed MSMAE framework incorporates a supervised attention-guided masking strategy (SAM) during the SFT phase, effectively directing the model’s attention toward lesion areas, thereby reinforcing the importance of SFT design in medical AI applications.

Despite its advantages, SFT also presents several challenges. Savage et al. [24] noted that when downstream tasks require high-level reasoning or involve complex semantic structures, SFT alone may lead to overfitting, catastrophic forgetting, or poor transferability. Thus, future research on SFT must focus on constructing more refined fine-tuning datasets, selecting suitable training strategies, and achieving a balance between retaining the general capabilities of pretrained models and enhancing domain-specific performance.

According to the systematic taxonomy by Han et al. [27], current Parameter-Efficient Fine-Tuning (PEFT) methods can be broadly categorized into four types: Additive Methods (e.g., Adapter, LoRA), Selective Methods (e.g., Bias Tuning, LayerNorm Tuning), Reparameterized Methods (e.g., Prefix Tuning, Prompt Tuning), and Hybrid Methods (e.g., Mix-Adapter, Unified Tuning). These strategies provide flexible design options tailored to different computational constraints and application scenarios.

In the medical domain, the effectiveness of PEFT has been well demonstrated. Dutt et al. [28] systematically evaluated 17 representative PEFT methods in medical imaging tasks. Their results show that under low-data regimes, PEFT approaches such as LoRA, SSF (Scale-Shift Feature), and Bias Tuning can achieve performance comparable to or even surpassing full fine-tuning while drastically reducing training resource requirements. They also observed that different PEFT strategies perform differently across model architectures (e.g., CNNs, ViTs) and task types (e.g., classification, generation), underscoring the importance of selecting appropriate fine-tuning techniques.

For medical image classification, Zu et al. [29] proposed Embedded Prompt Tuning (EPT), which pushes PEFT further toward efficiency. EPT directly modifies the input feature space without introducing large adapters or decoder modules and demonstrated strong transferability in cross-domain medical image classification tasks under few-shot learning settings.

In multi-task medical scenarios, Liu et al. [30] introduced the MOELoRA framework, combining the Mixture of Experts (MOE) architecture with LoRA-based tuning. This method assigns low-rank experts to individual tasks, effectively mitigating task interference and reducing fine-tuning costs, achieving state-of-the-art performance on the PromptCBLUE multi-task medical benchmark.

For medical question answering (Medical QA), Pandya [31] developed the peft-MedAware model, which fine-tunes only about 0.44% of parameters on top of the Falcon-1B base model while incorporating 4-bit quantization. This configuration significantly reduces both computational and storage requirements, and the model outperforms ChatGPT and Baize-Healthcare in producing more specific and accurate responses on the MedQuAD dataset.

Despite its advantages, Han et al. [27] noted that PEFT methods still face practical challenges, including limited cross-task transferability, increased inference latency, and the complexity of multi-adapter management. Future work must focus on both algorithmic innovation and system-level optimization to expand the practical applicability and scalability of PEFT approaches.

In contrast to these systems, the current study focuses on a highly specific and practical task—translating and summarizing echocardiography reports—under mid-tier hardware constraints. By employing parameter-efficient fine-tuning (QLoRA) on LLaMA-3.2 models, the proposed system achieves high-quality clinical text generation without requiring retrieval components or full-parameter tuning. This lightweight yet effective architecture makes it suitable for integration into real-world clinical workflows, particularly in resource-limited environments.

## 3. Results

### 3.1. Research Procedure

#### 3.1.1. System Architecture

**Figure 1:**
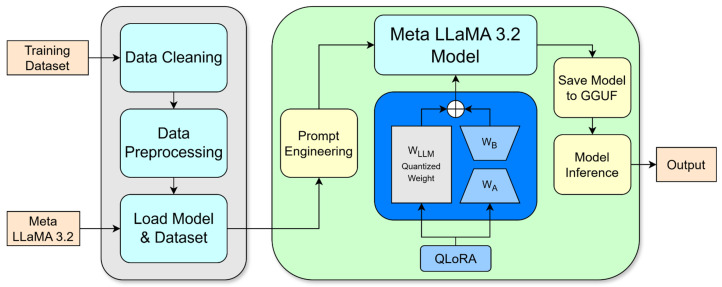
System architecture.

This study aims to construct a two-stage large language model (LLM) inference system capable of transforming specialized cardiac ultrasound reports written in English into clinically appropriate Traditional Chinese translations and, subsequently, into layperson-friendly summaries and preliminary recommendations. The system is designed to function under limited hardware conditions by integrating the Unsloth acceleration framework with the Quantized Low-Rank Adapter (QLoRA) fine-tuning technique. The overall system architecture, as illustrated in Figure 1, consists of four major components: dataset construction, model fine-tuning, dual-stage inference system, and performance evaluation.

In the dataset construction stage, two distinct datasets were prepared to support the two-stage generation tasks. For the translation task, a total of 250 real-world English cardiac ultrasound reports were annotated by cardiologists and translated into formal Traditional Chinese medical reports. This dataset was divided into 200 samples for training and 50 for validation. For the inference task, 150 samples were selected from the translated reports and annotated with public-facing summaries and recommendations tailored for non-medical readers; these were split into 120 training samples and 30 validation samples. Each sample was formatted into three structured fields: Instruction, Input, and Output. The instructions were specifically tailored for each task: “Translate the following cardiac ultrasound report into a formal Traditional Chinese medical report” for the translation task, and “Generate clinical inference based on the following Traditional Chinese ultrasound report” for the second-stage generation.

In the model fine-tuning stage, this study adopted Meta’s LLaMA-3.2 series as the base language models, selecting LLaMA-3.2-1B and LLaMA-3.2-3B-Instruct for training. The QLoRA technique was applied to enable 4-bit weight quantization and low-rank adaptation, significantly reducing memory usage during training. The Unsloth framework was incorporated to enhance efficiency through gradient checkpointing and efficient data packing. Two optimizer configurations were tested: paged_adamw_32bit and adamw_8bit, in order to evaluate their impact on training stability and generation quality under constrained GPU memory conditions.

The inference system was designed as a two-stage pipeline. The first-stage model receives an English cardiac ultrasound report and generates a formal Traditional Chinese medical report suitable for non-cardiologist physicians, aiding in diagnostic interpretation. The second-stage model then processes the first-stage output to generate simplified summaries and recommendations intended for the general public, improving accessibility and comprehension of medical information.

For model performance evaluation, this study used automated metrics including BLEU, ROUGE (ROUGE-1, ROUGE-2, ROUGE-L), METEOR, and Perplexity to assess the models’ translation accuracy, semantic consistency, linguistic fluency, and predictive capabilities. Comparative analysis was also conducted to examine the effect of different model sizes and optimizer settings on output quality and training efficiency, validating the feasibility and applicability of the proposed framework.

#### 3.1.2. Dataset Construction

To effectively fine-tune models for the tasks of translating and performing clinical inference on English echocardiography reports, this study constructed a specialized and structured dataset. The dataset covers both translation and inference tasks and was sourced from real-world echocardiographic reports. All data were curated and annotated by certified cardiologists to ensure high quality and semantic accuracy. The overall construction process includes data collection, expert annotation, format conversion, and dataset partitioning, as detailed below.

For the translation task, 250 representative English echocardiography reports were collected. These reports covered a wide range of clinical findings and linguistic expressions, such as valvular diseases, ventricular hypertrophy, chamber enlargement, and myocardial dysfunction. This diversity enables the model to learn a broad spectrum of cardiac pathologies and descriptive patterns. For the clinical inference task, a separate set of 150 examples was created based on the translated traditional Chinese reports, from which simplified clinical summaries and treatment suggestions were composed to simulate real-world diagnostic communication.

During the annotation phase, all outputs for both tasks were written and reviewed by professional cardiologists. The translation task outputs were formal traditional Chinese medical reports, aligned with clinical terminology and intended for non-cardiologist physicians. The inference task outputs were written in a more conversational and accessible style, providing layperson-friendly diagnostic summaries and general health advice, aimed at improving understanding for the general public.

The dataset follows an instruction fine-tuning format, where each example includes three key fields: Instruction, Input, and Output. The instruction for the translation task is standardized as follows: “Please translate the following English echocardiography report into a traditional Chinese medical report.” For the inference task, it is as follows: “Please generate a clinical summary based on the following Chinese echocardiography report.” The Input field contains either the original English report or the translated Chinese report, while the Output field contains the corresponding target text. All samples follow a customized Alpaca Prompt Template, and an EOS token is appended to each output to help the model recognize the end of the generated content.

The dataset was partitioned into training and validation sets as follows:

- Translation Task: 250 samples (200 for training, 50 for validation).
- Inference Task: 150 samples (120 for training, 30 for validation).

Samples were randomly assigned to each split while maintaining balance in disease types and report characteristics to support the model’s generalization ability across different clinical scenarios. Although labeled as a validation set, the 30-sample cohort used for the inference task. was strictly held out from the training process. No hyperparameter tuning or early-stopping decisions were based on this data, meaning it functionally serves as an independent test set to evaluate the model’s clinical generalization capabilities.

In terms of ethical considerations and data de-identification, all echocardiography reports utilized in this research were sourced from real-world clinical settings. To ensure patient privacy and comply with the Health Insurance Portability and Accountability Act (HIPAA) standards, a rigorous de-identification process was implemented:

- Removal of Identifiers: All personally identifiable information (PII), such as patient names, identification numbers, and specific dates of service, was manually removed by certified cardiologists before the dataset was provided for model training.
- Ethical Oversight: The study protocol was reviewed and supported by Taichung Veterans General Hospital, Taiwan R.O.C. (TCVGH-).
- Clinical Integrity: Despite de-identification, the structural and clinical integrity of the reports was maintained to ensure the models learned accurate cardiac pathology patterns.

Overall, this dual-stage dataset—professionally annotated and validated by cardiologists—ensures high clinical accuracy and semantic consistency. It provides a solid foundation for training translation and inference models and supports future expansion into other medical domains and clinical applications.

#### 3.1.3. Prompt Format Design Principles

In instruction-based fine-tuning frameworks, the instruction plays a critical role in guiding the model to understand the task objective. To this end, the prompt format used in this study is based on the Alpaca prompt template and was adapted to meet the specific requirements of the translation and summarization tasks. Each data sample includes three key components: Instruction (task description), Input (content to be processed), and Output (expected result).

The Instruction explicitly defines the task the model is expected to perform, such as “Please translate the following English echocardiography report into a formal Traditional Chinese medical report,” or “Based on the following Chinese medical report, generate a simplified and easy-to-understand summary and treatment suggestion.” The Input provides either the original report or the output from the preceding stage, while the Output contains the final response corresponding to the task requirements.

In terms of formatting, each sample uses the <s> [INST] and [/INST] tags to delimit the instruction block, followed by the input content. The section then transitions to the Output using the prompt “### Response:”. An End-of-Sequence (EOS) token is appended to the end of each sample to help the model identify the end of the generated text.

This prompt format offers several key advantages:

- It clearly separates the instruction, input, and output components, enabling the model to better understand and distinguish between their respective roles.
- It supports both translation and summarization stages, making it well-suited for dual-stage generation tasks.
- It enhances data consistency and readability, which improves learning effectiveness during fine-tuning and the quality of model inference.

#### 3.1.4. Data Preprocessing Procedure

To ensure the quality and consistency of the fine-tuning dataset, this study implemented a multi-layered data preprocessing procedure during the preparation stage. The process includes character cleaning, format normalization, end-of-sequence marking, and structural formatting, with the goal of systematically constructing high-quality training data that aligns with the requirements of instruction-based fine-tuning.

During the character cleaning and normalization stage, the original data were thoroughly examined to remove invisible control characters, incorrect line breaks, and non-standard encoded symbols. Chinese and English punctuation were also standardized. This process helped prevent parsing errors caused by formatting anomalies and improved input consistency and stability during model training.

For instruction standardization, consistent phrasing and formatting were applied to the Instruction fields of all samples to help the model better interpret the intended task during fine-tuning. Specifically, clear and specific phrasing, such as “Please translate the following English echocardiography report into a formal Traditional Chinese medical report” and “Based on the following Chinese medical report, generate a simplified summary and treatment suggestions for the general public,” was used uniformly. This standardization reduced semantic ambiguity across training samples, contributing to improved instruction comprehension and inference accuracy.

In the end-of-sequence token processing stage, an EOS token was appended to the end of each output segment. This design allowed the model to learn structural termination signals during training, enabling more natural and complete text generation at inference time. Although a relatively minor step, the EOS marking played a critical role in enhancing output fluency and controlling response length.

During batch formatting and data validation, all preprocessed samples were uniformly converted into the predefined Instruction-Input-Output format. Each sample was manually inspected for structural integrity and format correctness, including the completeness of instruction blocks, input text, and expected outputs, as well as the correctness of punctuation and end-of-sequence markers. Only data that passed validation were included in the final fine-tuning dataset to ensure the reliability and performance of model training.

Through the above preprocessing stages, this study successfully constructed a well-structured, format-consistent, and semantically clear dataset, establishing a solid foundation for subsequent dual-model fine-tuning tasks and effectively reducing the risk of training bias or inference errors caused by poor data quality.

#### 3.1.5. Dual-Model Comparison Strategy

This study compares the fine-tuning and inference results of two models with different parameter scales—LLaMA-3.2-1B and LLaMA-3.2-3B-Instruct—to analyze the impact of model size on the following aspects:

- Content completeness and terminology accuracy in translation tasks.
- Language fluency and information compression capability in summarization tasks.
- Resource consumption and training stability during the training stage.
- Generation speed and system responsiveness during the inference stage.

Through this dual-model experiment, the study not only evaluates the feasibility of deploying different model sizes under varying hardware constraints but also provides empirical insights for selecting appropriate model scales based on specific application scenarios, such as professional medical use or public-facing health communication.

#### 3.1.6. Training Batch and Step Configuration

To balance memory constraints and training stability, this study adopts the following settings for batch size and training steps:

- Per Device Batch Size: Set to 4.
- Gradient Accumulation Steps: Set to 4.
- Max Training Steps: Configured based on task type and model size as follows:
  – Translation Stage: 80 steps for LLaMA-3.2-1B, 100 steps for LLaMA-3.2-3B-Instruct.
  – Clinical Inference Stage: 140 steps for LLaMA-3.2-1B, 150 steps for LLaMA-3.2-3B-Instruct.

#### 3.1.7. Learning Rate and Weight Decay Settings

To enhance training stability during the initial phase and facilitate convergence, the following configurations were applied for learning rate and weight decay:

- Initial Learning Rate: Set to $5\times 10^{-4}$. This moderate value prevents instability caused by overly large updates early in training while maintaining a reasonable convergence speed.
- Scheduler Type: A linear scheduler was used. This strategy maintains a higher learning rate in the early stages of training and gradually decays it over time, enabling finer adjustments to model weights in later stages and improving the quality of the generated outputs.
- Weight Decay: Set to 0.01. This introduces mild regularization to suppress overfitting and improve generalization during inference.

#### 3.1.8. Precision and Memory Management Settings

To fully leverage the precision technologies supported by modern GPUs, this study applied the following optimizations for numerical precision and memory management:

- Precision Type:
  – If the hardware supports BF16 (Brain Floating Point 16-bit), it is prioritized for its balance between computational stability and acceleration efficiency.
  – If BF16 is not available, FP16 (Float16) mixed-precision training is used as an alternative.
- Dynamic Memory Management: Enabled in conjunction with the Unsloth framework, incorporating memory optimization techniques such as gradient checkpointing and efficient packing to further reduce peak VRAM usage.

#### 3.1.9. Hyperparameter Configuration

To ensure optimal learning performance under limited resource conditions, this study conducted a systematic design and tuning of hyperparameters, focusing on aspects such as LoRA adapter configuration, training batch setup, learning rate scheduling, precision management, and random seed control. This section provides a detailed explanation of the guiding principles and design considerations behind each hyperparameter setting. Table 1 shows the parameters used for LLaMA-3.2 models.

#### 3.1.10. Assessment of Physician-Authored

We invited seven senior physicians to evaluate the quality of the translations: one was a formal report written by a physician specializing in echocardiography, and the other was a computer-generated report. The reviewers completed a seven-question questionnaire to identify different aspects of the American Society of Echocardiography (ASE) recommended echocardiography reports (Table 2). The reviewers used a five-point rating scale to indicate their level of agreement.

Descriptive data are expressed as mean (standard deviation) and median (interquartile range). The Wilcoxon signed-rank test was used to compare the quality of ultrasound reports written by cardiologists with those generated by LLaMA Models. A *p*-value less than 0.05 was considered statistically significant for all analyses.

### 3.2. Experimental Results

#### 3.2.1. Training Curves of the Translation Model

**Figure 2:**
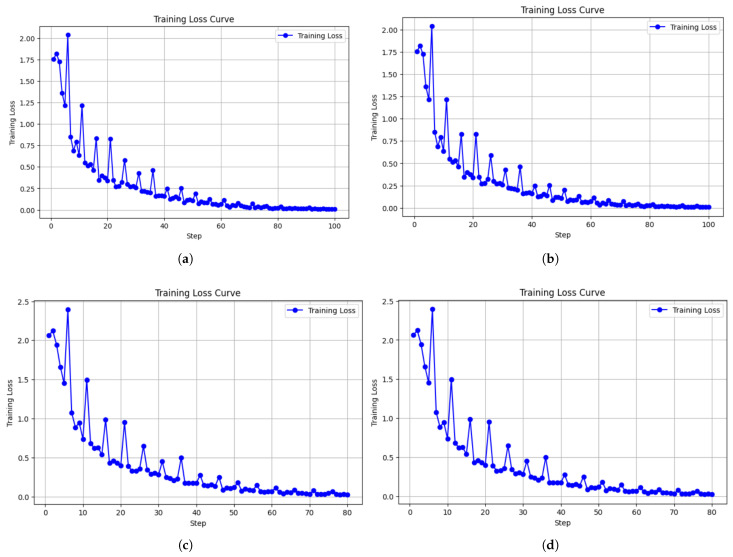
The training a loss over a different optimizer. (**a**) Training loss curve of the translation model (LLaMA-3.2-3B-Instruct with AdamW 8bit). (**b**) Training loss curve of the translation model (LLaMA-3.2-3B-Instruct with Paged AdamW 32bit). (**c**) Training loss curve of the translation model (LLaMA-3.2-1B with AdamW 8-bit). (**d**) Training loss curve of the translation model (LLaMA-3.2-1B with Paged AdamW 32bit).

This section presents the training loss curves of the translation model under four different combinations of model size and optimizer configuration. The models include LLaMA-3.2-3B-Instruct and LLaMA-3.2-1B, each fine-tuned using AdamW (8-bit) and Paged AdamW (32-bit). The training loss over time reflects the convergence behavior and stability of each configuration during the fine-tuning process. Figure 2a–d show the corresponding training loss curves.

Figure 2a illustrates the training curve of the LLaMA-3.2-3B-Instruct model with the AdamW 8-bit optimizer. The initial training loss is around 1.8, which drops rapidly within the first five steps, indicating the model quickly starts learning the task. Although some fluctuations are observed early on, the curve steadily decreases and plateaus after step 40. The final loss stabilizes below 0.05, demonstrating smooth and stable convergence throughout the training.

Figure 2b shows the same model trained with the Paged AdamW 32-bit optimizer. The initial loss also ranges from 1.7 to 2.0 and decreases quickly within the first 20 steps. Similar to the AdamW 8-bit configuration, the curve shows consistent convergence and stabilizes below 0.05. Despite minor fluctuations, the overall trend is smooth, and the model converges efficiently, suggesting that the 3B model performs robustly across both optimizer configurations.

In contrast, Figure 2c presents the training loss curve of the LLaMA-3.2-1B model using the AdamW 8-bit optimizer. The initial loss is slightly higher, around 2.0 to 2.4, and although the loss decreases rapidly in the first 10 steps, more significant oscillations are observed throughout training. During the middle phase (steps 20 to 50), the curve gradually stabilizes, but intermittent spikes are still noticeable, indicating higher sensitivity to batch variance and learning rate. Nevertheless, the model eventually converges with a final loss below 0.05, meeting the expected training goal.

Figure 2d shows the training curve of the LLaMA-3.2-1B model with the Paged AdamW 32-bit optimizer. The initial loss is comparable to that in Figure 2c, but the overall fluctuations are more intense. In the first 30 steps, the curve exhibits notable instability, including occasional upward spikes in the loss. Although the curve begins to stabilize after step 50 and ultimately converges, its irregular shape suggests that the 1B model with paged memory optimization is more sensitive to training conditions and may require more precise tuning of hyperparameters.

Overall, all four configurations successfully converge to a final loss below 0.05. However, the LLaMA-3.2-3B-Instruct model demonstrates superior training stability and convergence consistency regardless of the optimizer used. In contrast, the LLaMA-3.2-1B model shows greater sensitivity—especially when paired with the Paged AdamW optimizer—highlighting the need for more careful adjustment of training strategies and memory management when fine-tuning smaller models.

#### 3.2.2. Training Curves of the Inference Model

**Figure 3:**
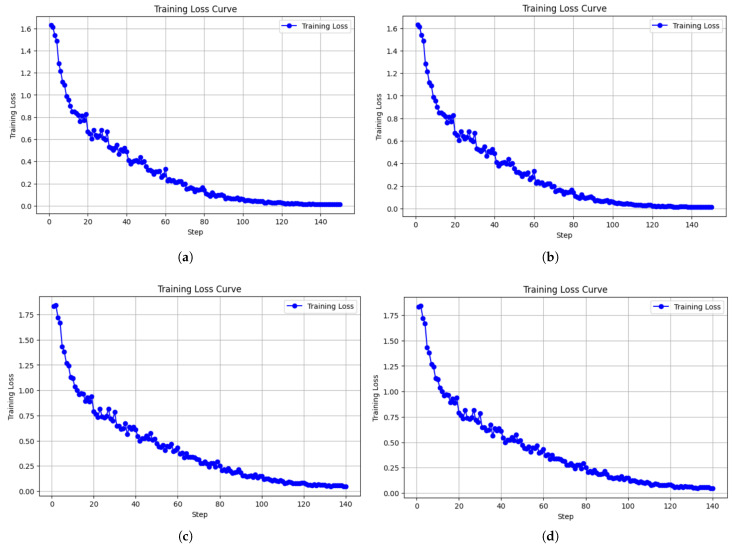
The training loss of the inference model. (**a**) Training loss curve of the inference model (LLaMA-3.2-3B-Instruct with AdamW 8bit). (**b**) Training loss curve of the inference model (LLaMA-3.2-3B-Instruct with Paged AdamW 32bit). (**c**) Training loss curve of the inference model (LLaMA-3.2-1B with AdamW 8-bit). (**d**) Training loss curve of the inference model (LLaMA-3.2-1B with Paged AdamW 32bit).

This section presents the training loss trends of four model configurations during the fine-tuning phase for the inference task, in order to evaluate their convergence speed and training stability. Figure 3a–d correspond to the following configurations: LLaMA-3.2-3B-Instruct with AdamW 8bit, LLaMA-3.2-3B-Instruct with Paged AdamW 32bit, LLaMA-3.2-1B with AdamW 8bit, and LLaMA-3.2-1B with Paged AdamW 32bit. Each model was trained for approximately 140 to 150 steps, with training loss recorded at each step as a key metric to assess learning efficiency and curve stability during inference fine-tuning.

Figure 3a shows the training loss curve of the LLaMA-3.2-3B-Instruct model using the AdamW 8-bit optimizer. The initial loss was around 1.6 and dropped rapidly to below 0.6 within the first 30 steps. The curve continued to decline smoothly without notable oscillations, and the final loss stabilized close to zero. This indicates excellent learning performance and stability, suggesting high compatibility between this model-optimizer pair and tasks involving semantic understanding and generation.

Figure 3b depicts the results of the same model with the Paged AdamW 32-bit optimizer. The initial loss was similar, around 1.6, and decreased to below 0.8 within the first 20 steps. Although the curve remained generally stable, minor fluctuations were observed in the middle stage of training, indicating that parameter updates under paged memory management introduced slight variability. Nevertheless, the final loss still converged to below 0.05, confirming that the 3B model achieved solid learning performance under both optimization settings.

Figure 3c presents the training curve of the LLaMA-3.2-1B model with the AdamW 8-bit optimizer. The initial loss was slightly higher, around 1.8, but showed a clear downward trend within the first 30 steps. While the overall trend was stable, more noticeable oscillations appeared between steps 40 and 80 compared to the 3B model. Still, the model successfully converged near zero, demonstrating that this configuration maintains acceptable learning performance even with a smaller model.

Figure 3d illustrates the training curve of the LLaMA-3.2-1B model with the Paged AdamW 32-bit optimizer. Although the initial loss was similar to the previous configuration, the curve exhibited greater instability throughout the training process. Significant fluctuations occurred, particularly between steps 40 and 70, reflecting increased sensitivity in smaller models to learning rate and parameter updates when using paged optimization strategies. Despite this, the final loss still decreased and approached zero, indicating the model’s acceptable fine-tuning potential under less stable conditions.

Overall, the LLaMA-3.2-3B-Instruct model demonstrated superior stability and convergence speed under both optimizer configurations, with AdamW 8-bit showing the smoothest loss curve. In contrast, while the LLaMA-3.2-1B model also achieved successful convergence, its training curves exhibited greater volatility, especially when paired with the Paged AdamW 32-bit optimizer. These results suggest that smaller models require more careful control over optimizer selection and training strategy when fine-tuning for inference tasks.

#### 3.2.3. Training Loss Curve

To analyze the convergence behavior and training efficiency of the proposed system, all fine-tuning experiments were conducted on Google Colab using a single NVIDIA Tesla T4 GPU with 14.741 GB of memory. Despite operating under mid-tier hardware conditions, the system successfully completed QLoRA-based fine-tuning for both LLaMA-3.2-1B and LLaMA-3.2-3B-Instruct models with all optimizer configurations.

In terms of GPU memory usage, the 3B model with the adamw_8bit optimizer reserved approximately 6.057 GB of VRAM, while all other configurations—including both 1B models and the 3B model with paged_adamw_32bit—reserved approximately 6.867 GB. These results confirm the practicality and memory efficiency of the adopted QLoRA and Unsloth-based fine-tuning strategy.

The total training time was also recorded to evaluate time efficiency. For the 1B models, translation fine-tuning took approximately 7 min, and inference fine-tuning ranged from 16 to 17 min. In contrast, the 3B models required longer durations: around 21 min for translation and 48 to 50 min for inference, depending on the optimizer used. These findings demonstrate that the complete dual-model fine-tuning workflow can be executed within roughly 1.5 h on a single T4 GPU, validating the deployability of the proposed approach under real-world resource constraints.

#### 3.2.4. Evaluation Results of Translation Models

This section presents the evaluation results of the four fine-tuned models applied to the translation task: LLaMA-3.2-3B-Instruct with AdamW 8-bit optimizer, LLaMA-3.2-3B-Instruct with Paged AdamW 32-bit optimizer, LLaMA-3.2-1B with AdamW 8-bit optimizer, and LLaMA-3.2-1B with Paged AdamW 32-bit optimizer. All models were evaluated using the same translation test dataset, and the metrics included BLEU, ROUGE (ROUGE-1, ROUGE-2, ROUGE-L), METEOR, and Perplexity, providing a comprehensive view of translation quality, fluency, and language modeling performance. Table 3 shows the performance comparison of LLaMA-3.2 Models with Different Optimizers.

Among all configurations, the LLaMA-3.2-3B-Instruct model with the AdamW 8-bit optimizer delivered the best overall performance. It achieved a BLEU score of 0.572, the highest among all models, indicating strong alignment between the generated outputs and the reference translations. ROUGE scores were also impressive, with ROUGE-1 at 0.975, ROUGE-2 at 0.922, and ROUGE-L at 0.963, reflecting excellent coverage and structural consistency. The METEOR score reached 0.758, demonstrating strong semantic matching and lexical diversity. Although its Perplexity was 4.236—not the lowest—it remained within a stable and acceptable range, indicating overall fluency.

The model with the same base architecture but using the Paged AdamW 32-bit optimizer showed slightly weaker results. Its BLEU score dropped to 0.527, noticeably lower than its 8-bit counterpart. ROUGE-1, ROUGE-2, and ROUGE-L were 0.893, 0.838, and 0.882, respectively, suggesting lower content alignment and sentence-level consistency. The METEOR score was 0.693, and the Perplexity was 4.762—the second highest among all models—indicating relatively weaker language modeling capability. These findings highlight the significant impact of optimizer memory strategies on generation quality, even when the model architecture remains the same.

The smaller LLaMA-3.2-1B model paired with the AdamW 8-bit optimizer still showed decent results. Its BLEU score was 0.508, slightly below both 3B models. ROUGE-1 and ROUGE-L reached 0.946 and 0.909, respectively, indicating good structural preservation and content relevance. ROUGE-2 scored 0.848, and the METEOR score was 0.679. However, its Perplexity was the highest among all configurations at 4.815, revealing relatively lower stability in language modeling.

The LLaMA-3.2-1B model with Paged AdamW 32-bit demonstrated a more balanced performance. It achieved a BLEU score of 0.516, outperforming the other 1B variant. ROUGE-1, ROUGE-2, and ROUGE-L reached 0.943, 0.883, and 0.923, respectively, reflecting solid semantic preservation and structural recovery. The METEOR score was 0.697—higher than its counterpart—indicating better word-level matching and semantic coherence. It also recorded the lowest Perplexity at 4.160, suggesting the strongest predictive performance across all configurations.

The LLaMA-3.2-3B-Instruct model with the AdamW 8-bit optimizer achieved the highest scores in BLEU, ROUGE, and METEOR, making it the most stable and consistent configuration for translation tasks in this study. Meanwhile, the LLaMA-3.2-1B model with the Paged AdamW 32-bit optimizer stood out in Perplexity, showing the best language prediction capability. These results suggest that both model size and optimizer choice significantly influence generation quality. Future model selection and training strategies should therefore consider a balanced trade-off between task requirements and hardware constraints.

#### 3.2.5. Evaluation Results of Inference Models

This section presents the evaluation of four inference model configurations: LLaMA-3.2-3B-Instruct with AdamW 8-bit optimizer, LLaMA-3.2-3B-Instruct with Paged AdamW 32-bit optimizer, LLaMA-3.2-1B with AdamW 8-bit optimizer, and LLaMA-3.2-1B with Paged AdamW 32-bit optimizer. All models were evaluated using the same test set, and their output quality and language modeling capability were assessed using BLEU, ROUGE (ROUGE-1, ROUGE-2, ROUGE-L), METEOR, and Perplexity metrics. Table 4 describes the performance Metrics for LLaMA-3.2 Models (1B vs. 3B).

Among the four models, LLaMA-3.2-3B-Instruct with AdamW 8-bit optimizer achieved the best performance. It obtained the highest BLEU score of 0.6890, indicating strong alignment between the generated content and reference outputs. Its ROUGE-1, ROUGE-2, and ROUGE-L scores were 0.8943, 0.8141, and 0.8596, respectively, reflecting excellent content coverage and structural consistency. The METEOR score was 0.7664, highlighting good semantic alignment and lexical diversity. The Perplexity score was 4.3015, indicating stable language fluency.

The model using LLaMA-3.2-3B-Instruct with Paged AdamW 32-bit optimizer showed slightly lower overall performance. While its BLEU score was slightly higher at 0.6949, its ROUGE-1, ROUGE-2, and ROUGE-L scores (0.8694, 0.7978, and 0.8478) were slightly lower. The METEOR score of 0.7709 was marginally better, but the model had the highest Perplexity (4.7604) among all configurations, indicating less stable language prediction.

The LLaMA-3.2-1B model with AdamW 8-bit optimizer performed less effectively. Its BLEU score was 0.4130, with ROUGE-1, ROUGE-2, and ROUGE-L scores of 0.7699, 0.6466, and 0.7006, respectively, reflecting limited semantic coverage and structural reconstruction. The METEOR score was only 0.5317, and the Perplexity score was 4.3996, suggesting lower language fluency.

The LLaMA-3.2-1B model with Paged AdamW 32-bit optimizer showed slightly better balance. Its BLEU score was 0.4118, with ROUGE-1, ROUGE-2, and ROUGE-L scores of 0.7659, 0.6280, and 0.6742, maintaining reasonable consistency in sentence alignment and structure. The METEOR score was 0.5450, and the Perplexity score was 4.2452, the second lowest among the four, suggesting relatively stable language modeling performance.

Large-scale models (3B) generally outperformed the smaller 1B models across quality metrics. The choice of optimizer also had a noticeable impact on performance. The AdamW 8-bit configuration offered a favorable trade-off between memory efficiency and generation quality. Meanwhile, the Paged AdamW 32-bit optimizer with 1B models presented a viable option in resource-constrained scenarios, delivering stable inference performance. These results suggest that model selection and training strategy should be tailored to task characteristics and system constraints.

#### 3.2.6. Assessment Scale of Physician-Authored

The *p*-values for Actionability, Clinical Interpretation, and Conclusion were all less than 0.05 (Table 5). This indicates that reports generated by LLaMA models go beyond mere text conversion and enhance the practical value of the reports in clinical decision-making. The *p*-values for Guideline Compliance and Completeness were very close to 0.05. This is typically due to the small sample size (*n* = 7), making it difficult to achieve extremely small *p*-values; however, the improvement is still clear considering the increase in the mean score.

#### 3.2.7. Frontend Interface

**Figure 4:**
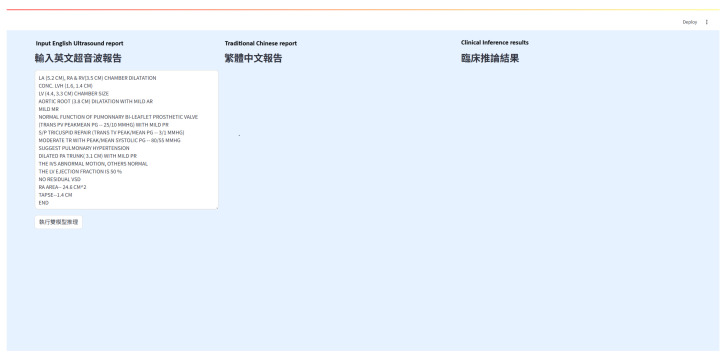
Three-column frontend interface initial state (before inference).

This study implemented a dual-model inference system with a user-friendly web interface built using Streamlit, deployed on Hugging Face Spaces. The interface consists of three horizontally arranged sections: the input area, the translation model output, and the clinical inference output. Users only need to paste or enter an English echocardiography report in the leftmost input box and click the “Execute Dual-Model Inference” button. The system sequentially triggers the translation and inference models and displays the results in real time (Figure 4).

**Figure 5:**
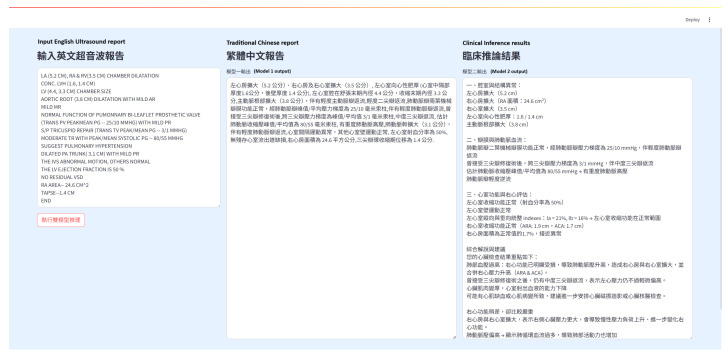
Three-column frontend interface after inference with input and output displayed.

The left section features an editable text area where users can input the original English report. The center section presents the output from the first-stage translation model, converting the English report into Traditional Chinese. The translated content is displayed in paragraph form for improved readability and professionalism. The right section shows the output of the second-stage clinical inference model, which analyzes the translated report and generates conclusions in four categories: abnormal structures, hemodynamic changes, ventricular function assessment, and overall clinical suggestions. This layout allows medical personnel to quickly grasp the key diagnostic points (Figure 5).

The interface is developed with Streamlit and deployed on Hugging Face Spaces, enabling web-based access without requiring any local installation. This setup reduces the deployment threshold and cost, and enhances reproducibility and openness for model development and demonstration. The interface design is clean and intuitive, making it accessible to healthcare professionals with varying technical backgrounds, and fulfilling the core objectives of clinical friendliness and real-time interactivity.

The Streamlit frontend includes a prominent clinical disclaimer. Every generated clinical summary is appended with a mandatory warning: The following content is for educational reference only. All diagnostic inferences must be reviewed and verified by a certified cardiologist before any clinical action is taken.

## 4. Discussion

While Section 3 provides a quantitative baseline of model performance, a comprehensive evaluation of a medical AI system requires a transition from linguistic metrics to clinical interpretation. Consequently, we include a qualitative error analysis in this Discussion section to explore the nuanced clinical risks—such as hallucinations and omissions—that automated scores may fail to capture, thereby providing a more rigorous assessment of the system’s safety profile.

### 4.1. Comparative Analysis and Clinical Safety Considerations

Based on the evaluation results from both the translation and summarization tasks, consistent performance trends were observed across different model and optimizer configurations. Among the external evaluation metrics (BLEU, ROUGE, METEOR), the combination of the LLaMA-3.2-3B model with the adamw_8bit optimizer achieved the best overall performance in both tasks. This indicates that the low-bit optimizer offers a good trade-off between memory efficiency and learning stability. In contrast, the performance gap between optimizers was relatively minor in the 1B model group, likely due to the limited model capacity.

In terms of task characteristics, the translation task yielded higher BLEU scores overall, suggesting that the fixed structure and frequent phrase patterns in medical reports made n-gram matching easier for the models. On the other hand, the summarization task involved semantic reorganization and content compression, making ROUGE-L and METEOR more suitable for evaluating model effectiveness. Notably, higher METEOR scores in summarization reflect the model’s ability to capture stemmed variations and synonym substitutions, indicating better semantic-level robustness.

In addition, Perplexity was used as a supplementary internal metric to evaluate the fluency of language modeling. The trends in Perplexity were consistent with those of the external metrics, confirming that the fine-tuning process improved both generation performance and language coherence. It is worth noting that the 3B model outperformed the 1B model in terms of Perplexity, demonstrating the positive impact of model size on next-token prediction capability.

For preliminary qualitative error analysis, an informal review of validation samples identified three primary types of clinically relevant errors that automated metrics failed to penalize:

- Over-Generalization: In some cases, the inference model provided broad health advice (e.g., “regular follow-up”) while omitting specific hemodynamic details present in the source translation.
- Terminology Simplification: While the goal was layperson accessibility, certain technical nuances regarding valvular regurgitation were simplified to the point of losing specific clinical grading (e.g., “mild” vs. “moderate”).
- Hallucination of Descriptive Adjectives: Rare instances were noted where the model added descriptive adjectives (e.g., “stable”) to a diagnosis that were not explicitly stated in the source English report, highlighting the need for stricter safety guardrails.

Table 6 describes the examples of clinically relevant qualitative errors.

To mitigate the risks associated with AI-generated medical advice, the system can adopt a “Human-in-the-Loop” philosophy.

- Intended Audience: The second-stage clinical inference is designed as an educational tool to improve health literacy among non-specialist physicians and the general public.
- Non-Actionable Guidance: The model’s prompts were standardized to prioritize the explanation of abnormal structures and hemodynamic changes over direct medical prescriptions.
- Clinical Verification: Every generated report includes a prominent warning that the content is for reference only and must be reviewed by a certified cardiologist before any clinical action is taken.

### 4.2. Ethical Implications and Clinical Safety

The generation of clinical summaries and preliminary treatment suggestions introduces critical ethical and safety considerations that must be addressed before real-world deployment [1,6].

#### 4.2.1. Risk of Medical Hallucinations

Large language models (LLMs), including the LLaMA-3.2 variants used in this study, are susceptible to “hallucinations”—the generation of plausible-sounding but factually incorrect information [6,12]. In the context of echocardiography, this could manifest as the fabrication of non-existent symptoms or the misreporting of vital cardiac parameters, such as ejection fraction percentages [12,24]. While our training loss curves show stable convergence, automated metrics like BLEU do not adequately penalize these high-stakes factual or clinical errors [12].

#### 4.2.2. Risk Mitigation and Safety Constraints

To minimize the impact of such errors, the following mitigation strategies are integrated into the system’s design [6,12]:

- Knowledge-Grounded Reasoning: Future iterations will explore integrating medical knowledge graphs to ground model outputs in clinical truth [12,15].
- Safety Guardrails: We have implemented standardized prompts that prioritize the explanation of abnormal structures over direct medical prescriptions to reduce actionable risk [12].
- Transparency: Every generated report includes a prominent warning that the content is for educational reference only and must be reviewed by a certified cardiologist [6,11].

#### 4.2.3. Intended Use: Decision Support vs. Autonomous Advice

It is essential to define the Intended Use of this system as a Clinical Decision Support System (CDSS) rather than an autonomous medical agent [6,11]:

- Decision Support (Augmentation): The system is designed as an educational tool to improve health literacy and assist in documentation for non-specialist physicians [8].
- Autonomous Use (Prohibited): The system is not intended for fully autonomous clinical use, as current LLMs cannot replace the nuanced clinical reasoning and ethical judgment of a human physician [6,12]. Final diagnostic responsibility remains solely with the human clinician.

#### 4.2.4. Multi-Layered Safeguards Against Medical Misinterpretation

To prevent the system’s outputs from being misused as autonomous medical advice, we have implemented a three-tier safeguard architecture:

- Technical Safeguards (Prompt Engineering): The LLaMA models are fine-tuned with strict system instructions that prioritize structural explanation over actionable prescription. For instance, the model is directed to describe a severely reduced ejection fraction and its hemodynamic implications rather than suggesting specific heart failure medications.
- Operational Safeguards (Human-in-the-Loop): The workflow is designed such that the clinical summary remains in a draft state within the Streamlit interface until a qualified clinician reviews and validates the findings. This ensures that the ultimate diagnostic responsibility and ethical judgment remain with a human physician.
- Linguistic Safeguards (Framing): The second-stage inference model utilizes hedging and conditional phrasing to clarify the probabilistic nature of the AI’s output. Every summary is dynamically appended with a localized warning stating that the content is for health literacy purposes only and does not constitute a doctor-patient relationship.

## 5. Limitations and Future Directions

### 5.1. Medical Report Prosessing

While this study demonstrates the technical feasibility of using parameter-efficient fine-tuning (PEFT) for medical report processing, several limitations must be acknowledged:

- Sample Size and Generalization: The datasets used for fine-tuning—250 samples for translation and 150 for inference—are relatively small. Although QLoRA and weight decay were employed to mitigate overfitting, the model’s ability to generalize to rare cardiac pathologies or highly non-standard report formats remains unverified. Smaller models, specifically the 1B variant, already exhibited increased sensitivity and training instability on this limited data.
- Risk of Recommendation Safety: The second-stage model provides preliminary clinical recommendations. Without a rigorous safety framework or a human-in-the-loop verification process, there is a risk that laypersons may over-rely on AI-generated advice.

### 5.2. Methodological Benchmarking and Comparative Constraints

While this study successfully implements a resource-efficient fine-tuning pipeline, it is subject to the following benchmarking limitations:

- Comparison of PEFT Strategies: The study exclusively utilized QLoRA due to its ability to stabilize training for 1B and 3B models on a single mid-tier GPU (NVIDIA Tesla T4). Direct experimental comparisons with other PEFT categories, such as Additive Methods (Standard LoRA, Adapters) or Reparameterized Methods (Prefix Tuning), were not conducted within this hardware-constrained scope.
- Zero-shot/Few-shot Benchmarking: Performance was not benchmarked against commercial or general-purpose LLMs in zero-shot or few-shot settings. While such models demonstrate strong general reasoning, their application in specialized cardiology tasks often faces challenges regarding clinical accuracy and data privacy.
- Optimization Frameworks: The results are specific to the Unsloth acceleration framework. Future research could explore whether other optimization backends provide different trade-offs in inference latency and semantic quality.

## 6. Conslusions

This study successfully developed a dual-stage inference system for translating and summarizing English echocardiography reports using LLaMA-3.2 models (1B and 3B), QLoRA, and Unsloth. Comprehensive evaluation using BLEU, ROUGE, METEOR, and Perplexity metrics identified the 3B model with the AdamW 8-bit optimizer as the superior configuration, delivering optimal semantic alignment and fluency. While the 1B model demonstrated viability for memory-constrained environments, the 3B variant ensures the highest accuracy for clinical deployment. The system, accessible via a Hugging Face-hosted Streamlit interface, provides a seamless workflow from raw report input to translated clinical inference. Clinically, this framework addresses critical communication gaps. By ensuring terminological precision, it assists general practitioners and critical care teams in rapidly interpreting complex cardiac parameters, reducing reliance on specialist consultations. Furthermore, the generation of patient-centric summaries enhances health literacy and facilitates shared decision-making. Future work will focus on three key expansions: integrating echocardiographic imaging for multimodal visual-language understanding; applying advanced quantization (e.g., GPTQ, AWQ) and dynamic fine-tuning to further reduce latency; and generalizing this dual-model architecture to other medical domains such as radiology and oncology. This study serves as a foundational step toward intelligent, interpretable, and widely accessible clinical language generation systems. Future work will prioritize the collection of a larger, independent held-out test set from diverse clinical environments to verify the system’s robustness and clinical generalizability.

## Figures and Tables

**Table 1:** Fine-tuning hyperparameters used for LLaMA-3.2 models.

Hyperparameter	Value
Pretrained Model	LLaMA-3.2-3B-Instruct/LLaMA-3.2-1B
Fine-Tuning Method	QLoRA (with Unsloth framework)
Optimizer	AdamW 8bit/Paged AdamW 32bit
Batch Size	4
Gradient Accumulation	4
Max Training Steps	Translation: 100 (3B)/80 (1B)
	Inference: 150 (3B)/140 (1B)
Learning Rate	$5\times 10^{-4}$
Weight Decay	0.01
LR Scheduler	Linear
Logging Interval	Every 1 step
Random Seed	3407
LoRA Rank	16
LoRA Alpha	32
Target Modules	q_proj, k_proj, v_proj, o_proj,
	gate_proj, up_proj, down_proj
Quantization Bits	4-bit

**Table 2:** Assessment scale for evaluating the quality of echocardiogram reports.

Item	Description	Score (1–5)
Completeness	This translation report covers all the important components of the heart.	
Accuracy	The accuracy of quantitative and qualitative descriptions in the report.	
Guideline adherence	The report follows the standard terminology established by the American College of Ultrasound Cardiology and the European Society of Cardiovascular Imaging, and the standard measurement methods and units are correct.	
Internal consistency	The reports should be consistent in pathophysiology, and all parameters should reflect the same physiological condition.	
Conclusion	Instead of simply repeating the numbers in the data sheets, the report should answer clinically relevant questions and clearly identify potential underlying causes.	
Clinical interpretation	The report goes beyond mere description; it also answers questions such as, What is the primary diagnosis? What are the hemodynamic implications? How severe is the condition?	
Actionability	This report can guide clinical decision-making: it includes follow-up recommendations, whether further imaging studies (transesophageal echocardiography, CT, MRI) or surgical/interventional treatment are needed.	

**Table 3:** Performance comparison of LLaMA-3.2 models with different optimizers.

LLaMA Model	Optimizer	BLEU	R-1	R-2	R-L	MET	PPL
3.2-1B	AdamW 8bit	0.508	**0.946**	0.848	0.909	0.679	4.815
	Paged AdamW 32bit	**0.516**	0.943	**0.883**	**0.923**	**0.697**	**4.160**
3.2-3B-Instruct	AdamW 8bit	**0.572**	**0.975**	**0.922**	**0.963**	**0.758**	**4.236**
	Paged AdamW 32bit	0.527	0.893	0.838	0.882	0.693	4.762

Note: R-1 = ROUGE-1, R-2 = ROUGE-2, R-L = ROUGE-L, MET = METEOR, PPL = Perplexity.

**Table 4:** Validation performance metrics for LLaMA-3.2 inference models.

LLaMA Model	Optimizer	BLEU	R-1	R-2	R-L	MET	PPL
3.2-1B	AdamW 8bit	0.413	0.770	0.647	0.701	0.532	4.400
	Paged AdamW 32bit	0.412	0.766	0.628	0.674	0.545	4.246
**3.2-3B-Instruct**	**AdamW 8bit**	**0.689**	**0.894**	**0.814**	**0.860**	**0.766**	**4.301**
	Paged AdamW 32bit	0.695	0.869	0.798	0.848	0.771	4.760

Note: Results represent validation performance (*n* = 30). Reported “Inferences” are intended for clinical decision support and educational guidance only. All outputs require verification by a certified cardiologist to ensure patient safety.

**Table 5:** Comparison of the assessment scale between physician-authored and LLaMA models proposed notes.

	Physician-Authored		LLaMA Models Proposed		
**Domain**	**Mean ± SD**	**Median (IQR)**	**Mean ± SD**	**Median (IQR)**	**p-Value**
Completeness	3.6±0.5	4.0 (3.0–4.0)	4.4±0.5	4.0 (4.0–5.0)	0.06
Accuracy	3.9±0.4	4.0 (4.0–4.0)	4.3±0.5	4.0 (4.0–4.5)	0.08
Guideline adherence	3.7±0.5	4.0 (3.5–4.0)	4.4±0.5	4.0 (4.0–5.0)	0.06
Internal consistency	3.6±0.5	4.0 (3.0–4.0)	4.0±0.6	4.0 (4.0–4.0)	0.08
Conclusion	3.4±1.0	3.0 (3.0–4.0)	4.4±0.5	4.0 (4.0–5.0)	0.02 *
Clinical interpretation	2.9±0.7	3.0 (2.5–3.0)	4.7±0.5	5.0 (4.5–5.0)	0.02 *
Actionability	1.9±0.7	2.0 (1.5–2.0)	4.6±0.5	5.0 (4.0–5.0)	0.02 *

Abbreviation: IQR, interquartile range; LLaMA, large language model Meta AI; SD, standard deviation. Note: Wilcoxon signed-rank test. * p<0.05.

**Table 6:** Examples of clinically relevant qualitative errors in inference outputs.

Error Type	Example of Model Output	Clinical Concern
Over-Generalization	“Maintain a healthy lifestyle and follow up regularly with a physician.”	Omits critical hemodynamic details such as specific valvular pressure gradients.
Terminology Simplification	“The patient has a leaky heart valve.”	Fails to provide the specific grading (e.g., mild, moderate, or severe) necessary for surgical planning.
Hallucination of Adjectives	“The patient exhibits stable left ventricular function.”	Adds the descriptor “stable” which was not explicitly stated in the objective measurements of the source report.

Note: These examples were identified during a preliminary manual review of the 30-sample validation cohort used for the inference.

## Data Availability

The data presented in this study are available on request from the corresponding author due to ethical restrictions.
